# Robust Laser‐Induced Graphene‐Boron‐Doped Diamond Nanowall Hybrid Nanostructures with Enhanced Field Electron Emission Performance for Microplasma Illumination Devices

**DOI:** 10.1002/smsc.202400430

**Published:** 2025-04-21

**Authors:** Mohsen Khodadadiyazdi, Mateusz Ficek, Maria Brzhezinskaya, Shradha Suman, Salila Kumar Sethy, Kamatchi Jothiramalingam Sankaran, Bartłomiej Dec, Mattia Pierpaoli, Sujit Deshmukh, Miroslaw Sawczak, William A. Goddard, Robert Bogdanowicz

**Affiliations:** ^1^ Institute of Nanotechnology and Materials Engineering Faculty of Applied Physics and Mathematics Gdańsk University of Technology 11/12 Narutowicza St. 80‐233 Gdańsk Poland; ^2^ Faculty of Electronics, Telecommunications, and Informatics Gdańsk University of Technology 11/12 Narutowicza St. 80‐233 Gdańsk Poland; ^3^ Main Department Scientific Equipment Development Helmholtz‐Zentrum Berlin für Materialien und Energie Hahn‐Meitner‐Platz 1 14109 Berlin Germany; ^4^ CSIR‐Institute of Minerals and Materials Technology Bhubaneswar Odisha 751013 India; ^5^ Academy of Scientific and Innovative Research (AcSIR) Ghaziabad 201002 India; ^6^ Central European Institute of Technology Brno University of Technology Purkyňova 123 61200 Brno Czech Republic; ^7^ Szewalski Institute of Fluid‐Flow Machinery Polish Academy of Sciences Fiszera 14 Gdansk Poland; ^8^ Materials and Process Simulation Center California Institute of Technology 1200 East California Blvd. California 91125 USA

**Keywords:** ab‐initio simulations, edge‐rich diamond/graphene nanostructures, electron field emission, flexible cathode, laser‐induced graphene

## Abstract

This investigation introduces a scalable fabrication method for laser‐induced graphene (LIG)‐boron‐doped diamond nanowall (BDNW) hybrid nanostructures, designed for field electron emission (FEE) cathode materials in microplasma illumination (μPI) devices. The two‐step process involves fabricating BDNWs via microwave plasma‐enhanced chemical vapor deposition, followed by drop‐casting BDNW dispersion onto polyimide foils to create LIG‐BDNW hybrid nanostructures. Topographic studies reveal that BDNWs on LIG boosts surface area and prevent graphene restacking. High‐resolution transmission electron microscopy confirms precise BDNW decoration, creating sharp edges and high porosity. The effects of boron and nitrogen dopants, highlighted by Raman spectroscopy, are corroborated by near‐edge X‐ray absorption fire structure and X‐ray photoelectron spectroscopies. The hybrid nanostructures exhibit high electrical conductivity and superior FEE properties, with a low turn‐on field of 2.9 V μm^−1^, a large FEE current density of 3.0 mA cm^−2^ at an applied field of 7.9 V μm^−1^, and a field‐enhancement factor of 5,480. The hybrid nanostructures demonstrate an exceptionally low breakdown voltage of 320 V and a plasma current density of 9.48 mA cm^−1^ at an applied voltage of 550 V. Ab‐initio calculations of the electronic structure further support the experimental findings of these diamond–graphene hybrids, underscoring their potential in advanced electronic applications.

## Graphical abstract

A facile procedure is developed for creating laser‐induced graphene (LIG)‐boron‐doped diamond nanowall (BDNW) nanostructures on flexible polyimide films. Although BDNW incorporation has resulted to increased defect density in the LIG, the obtained nanostructures benefit from enhanced electrical conductivity and specific surface area. When utilized in plasma illumination devices, both plasma current density and breakdown voltage are very high.

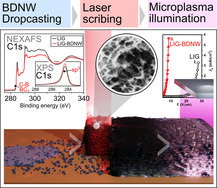

## 1 Introduction

Microplasma, which is referred to as miniaturized plasma, confined within sub‐millimeter gaps, is usually generated at low temperature and atmospheric pressure.^[^ ^1^ ^]^ As a class of nonthermal plasma, microplasma is under strong nonequilibrium conditions, possesses high power density and a multi‐temperature environment with highly reactive species, and a strong gradient is present. It can provide an extreme environment for the synthesis of materials that are far‐from‐equilibrium, such as kinetically trapped structures and metastable morphologies.^[^ ^2^ ^]^ This has resulted in unique applications of microplasma in the synthesis of advanced materials and the development of special devices.^[^ ^3^ ^]^ Extensive research and development on microplasmas, has resulted in numerous application such as flat displays, light sources, polymer modifications, and microelectronic and optoelectronic devices.^[^ ^4^ ^]^ However, it is not only plasma that affects the micro‐/nanostructure of synthesized materials but also the composition and microstructure of the constituent materials of cathodes, affecting the microplasma illumination (μPI) properties of devices. μPI can be utilized as an excitation or light source in a variety of applications ranging from analytical chemistry to plasma displays.^[^ ^5^, ^6^, ^7^ ^]^ The materials, fromwhich cathodes for μPI devices are made of, must have a high secondary electron emission coefficient (SEEC), and the ability to withstand the harsh environmental conditions of plasma. In this regard, carbon allotrope‐based materials, such as diamond and graphene, have been widely studied for μPI applications.

Diamond, containing *sp* ^3^ hybridized carbon atoms in a three‐dimensional (3D) structure, benefit from unique features such as high mechanical properties and exceptional thermal conductivity. However, inducing electrical conductivity, via doping, further improves its favorable properties and functionalities, making it a promising electrode candidate in diverse fields such as sensing and energy applications.^[^ ^8^, ^9^ ^]^ Boron‐, phosphorous‐, and nitrogen‐doping is normally used to induce local electron deficiency or electron abundance corresponding to p‐type or n‐type semiconducting behavior, respectively. Based on morphology, diamond nanostructures are categorized as microcrystalline diamond (MCD), nanocrystalline diamond (NCD), or ultrananocrystalline diamond (UNCD). Previous results have revealed that, when used as cathodes in μPI devices, regardless of morphologies, these diamond nanostructures show similar SEECs.^[^ ^10^ ^]^ In order to improve the μPI behavior of diamond nanostructures, it is important to understand the microstructure‐doping features‐physicochemical properties–μPI relationship for diamond nanostructures. Microwave plasma‐enhanced chemical vapor deposition (MWPECVD) is a popular strategy for making micro‐ and nanostructured diamonds that are doped with trace amounts of elements from group 3 or 5 in the periodic table. Controlling the nucleation and growth of diamond nanostructures is feasible through controlling the composition of reactive gases, substrate temperature, and time in the MPECVD process. Previous studies have shown that, for CVD, the composition of the reactive gases and substrate temperature affect the morphology, grain structure, and final properties of the produced diamond nanostructures. This is because the chemical makeup of reactive species (e.g., ions and radicals) in the CVD environment is impacted by the composition of reactive gases. Furthermore, the substrate temperature affects both adsorption/desorption kinetics of reactive species and kinetics of carbon atom rearranging, for example, *sp* ^3^‐to‐*sp* ^2^ transformation, and grain growth.^[^ ^11^ ^]^ The competitive effects of the kinetics of adsorption, and the kinetics and thermodynamics of the formation/cleavage of carbon–carbon bonds, and stacking behavior of carbon macromolecular structures via physical interactions, dictates the final ratio of various carbon allotropes and morphology of the obtained diamond nanostructure.^[^ ^12^ ^]^

It was previously found that the presence of nitrogen ions can trigger the formation of the graphitic phase, while phosphorous ions improve the electrical conductivity at the interface. The overall effect is increasing the electrical conductivity, resulting in enhanced μPI features and improved lifetime stability.^[^ ^13^ ^]^ In most previous studies, nanostructured diamond films have been studied as a cathode material and the main goal has been increasing the electrical conductivity and increasing the number of illumination sites through a doping process and introducing gases that induce the graphitic phase at grain boundaries. However, a new strategy seeks to incorporate diamond nanostructures into other conductive materials to make hybrid nanostructures with improved μPI behavior. Laser‐induced graphene (LIG) benefiting from a highly 3D porous structure, high electrical conductivity, and favorable field electron emission (FEE) have been considered as a promising host for diamond nanostructures. In addition, LIG can be rapidly manufactured on flexible substrates, which makes it favorable from an economic point of view.^[^ ^14^, ^15^ ^]^ Recent studies highlight the potential for facile modification of raw materials, such as simple coatings on polyimide films, to overcome the electrochemical limitations of conventional LIG, enabling the fabrication of nanostructure‐decorated LIG hybrids and enhancing the performance of LIG‐based devices such as energy storage devices.^[^ ^16^, ^17^, ^18^ ^]^ Accordingly, hybrid nanostructures based on diamond nanostructures with attractive μPI properties hosted by porous and conductive LIG were speculated to increase the μPI characteristics of diamond nanostructures.

In this investigation, LIG‐boron‐doped diamond nanowall (BDNW) hybrid nanostructures were designed to create flexible and robust field electron emission (FEE) cathode materials. The LIG‐BDNW hybrids were fabricated using a combination of the MWPECVD process to form BDNWs, followed by a rapid lasing process to induce 3D graphene‐diamond topographies. When used as cathode materials for μPI devices, these hybrids exhibited superior FEE characteristics. The effects observed in the 3D hybrid nanostructures were evaluated using Raman spectroscopy, SEM, TEM, NEXAFS spectroscopy, and XPS studies, and were supported by DFT simulations.

## 2 Results and Discussion

### 2.1 Manufacturing Process and Microstructural Features

**Figure 1:**
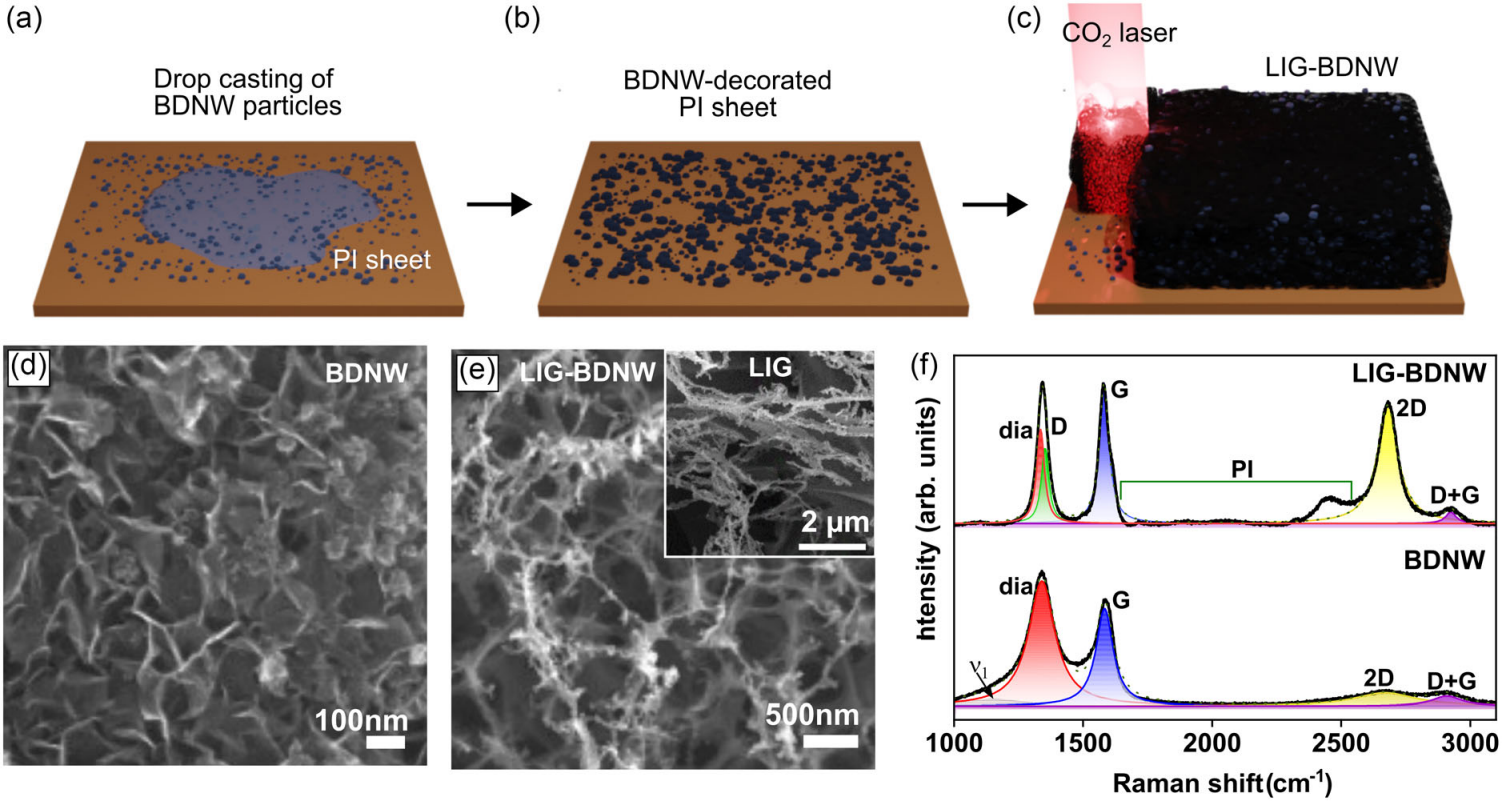
Fabrication and characterization of LIG‐BDNW hybrid nanostructures. a) Drop casting a dispersion of BDNW on PI foils. b) Solvent evaporation under environmental conditions to obtain BDNW‐decorated PI foils. c) Laser scribing process to prepare LIG‐BDNW hybrid nanostructures. FESEM micrograph of d) BDNW nanoparticles and e) LIG‐BDNW hybrid with the inset showing pristine LIG. f) Raman spectra for I. BDNW and II. LIG‐BDNW hybrids.

A MWPECVD process, in combination with a laser scribing process, was proposed to synthesize novel hybrid nanostructures. In the first step, MWPECVD was utilized to grow BDNW nanostructures, for which a detailed procedure is presented in the experimental section. The second step included drop‐casting BDNW dispersions on a flexible polyimide (PI) foil to obtain BDNW‐decorated PI foils, followed by laser scribing to obtain hybrid nanostructures, as schematically illustrated in **Figure** **1**a–c.

**Figure 2:**
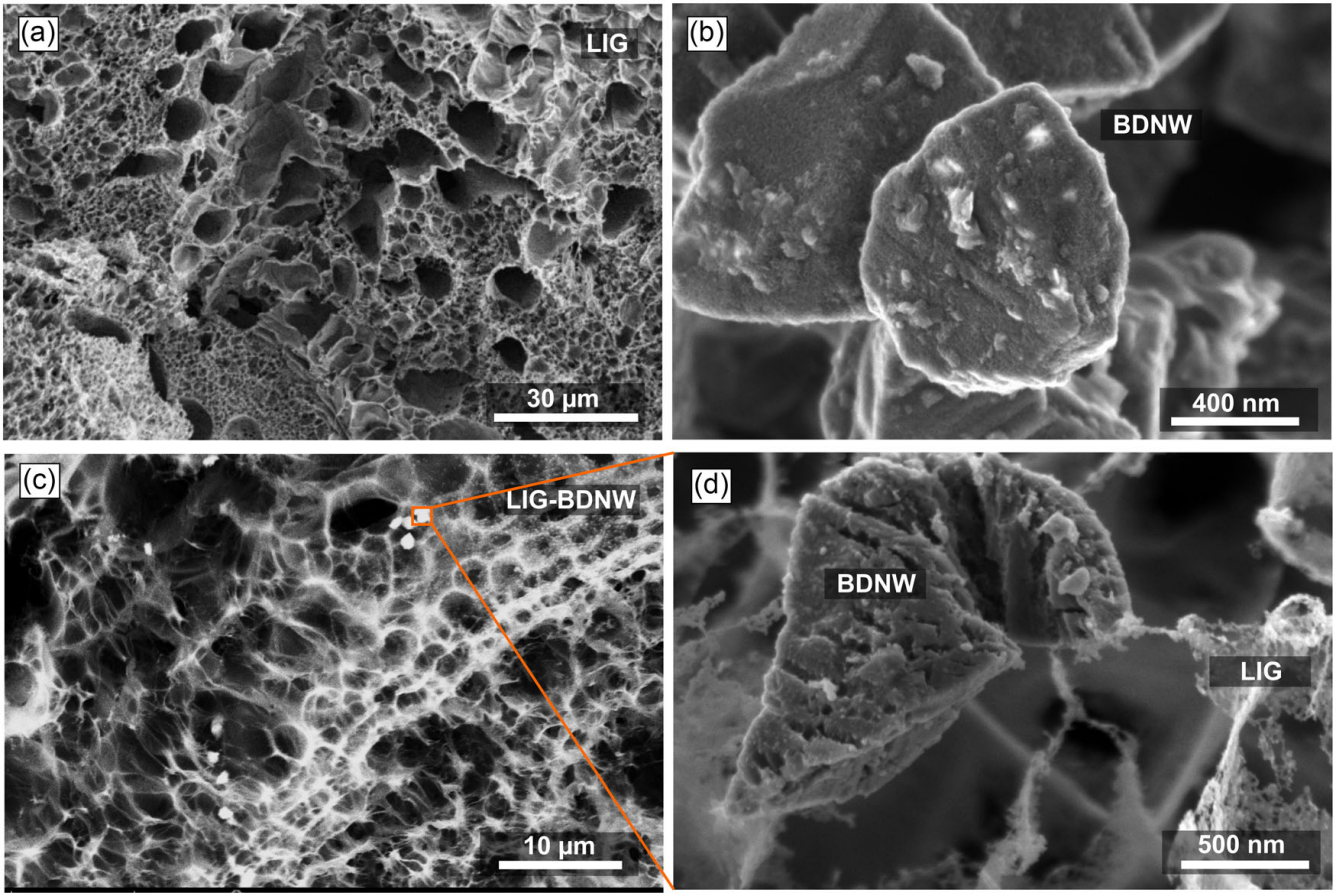
SEM micrographs of nanostructures. a) SEM top view of LIG showing a porous 3D morphology. b) SEM image of BDNW particles with dimensions ranging ≈1–2 μm. c) SEM image of LIG‐BDNW where BDNW (white spots) are distributed across the LIG network. d) magnified view of the circular region in part c, displaying the attachment of BDNW particles with the LIG nanoplatelets.

Prior to drop casting, BDNW was dispersed in dimethyl sulfoxide (DMSO) with the aid of ultrasonication for ≈30 min to obtain BDNW dispersions at a concentration of 2 mg mL^−1^. After drying at room temperature (RT), diamond‐decorated PI foils were subjected to laser scribing using a commercial CO_2_ laser. Laser irradiation of PI foils rapidly increases the local temperature, peaking at 2000 °C, resulting in polymer bond dissociation, creation of reactive species, and formation of stable graphene nanostructures.^[^ ^19^ ^]^ This intense thermal shock at a sub‐millisecond time scale prompts the formation of carbonized steam. At the same time, the photothermal reaction triggers the release of various gases, including oxygen (O_2_), carbon monoxide (CO), carbon dioxide (CO_2_), methane (CH_4_), and nitrogen (N_2_), at temperatures over 700 °C.^[^ ^20^, ^21^, ^22^ ^]^ Liberation of these gaseous by‐products plays a crucial role in the formation of complex 3D porous interconnected structure of LIGs, as shown in **Figure** **2**a. This image shows the uneven distribution of pores in LIG structure originating from the rapid but haphazard release of hot by‐product gases from underneath thermochemical reactions happening on the polymer substrate. Figure 2b shows SEM micrographs of BDNW particles with random shapes and various sizes, from submicrons to a few microns.

BDNW particles are characterized by their sharp edges induced by dopants and the faceted morphology originating from diamond cores, with individual particle dimensions varying ≈1–2 μm. In the case of the LIG‐BDNW hybrids, effective adhesion and uneven distribution of BDNW particles onto the surface of the 3D LIG network are observable in Figure 2c. It seems that the drop‐casting and drying procedure result in a nonuniform distribution as a result of incomplete dispersion and deagglomeration during ultrasonication, and surface tension‐induced effects such as the coffee‐ring phenomenon. It is also possible that gas release and the development of the 3D structure of the LIG lead to the displacement of BDNWs. Upon closer examination under high magnification, as illustrated in Figure 2d, the micron‐sized BDNW particles become more prominently visible, exhibiting that BDNW particles are encircled by some nanofibrous LIG structures. Various morphological transitions as a result of laser fluence were previously reported.^[^ ^23^ ^]^ However, in the current work, since the laser fluence is fixed, enhanced laser absorbance by BDNW is likely the responsible mechanism of such morphological transitions in the vicinity of diamond nanowalls. Transformation from *sp* ^2^ to *sp* ^3^ hybridized carbons at the interface of BDNW particles‐LIGs simulates inorganic Janus nanostructures, in which each face possesses distinct physical properties, resulting in enhanced functionality of these anisotropic hybrid nanostructures.^[^ ^24^ ^]^ The heterostructure featuring such a decoration of BDNW over LIG serves not only to enhance the surface area but also effectively prevents the self‐restacking of graphene sheets.

The laser scribing process does not involve any additional processing steps or chemicals, and easily produces patterned and highly porous graphene with repeatable quality at high speed. This indicates that LIG formation is a green and economical procedure for making graphene‐based nanostructures. In fact, LIG and flash graphene seem to be more economically favorable compared to other available techniques for graphene synthesis.^[^ ^14^, ^25^ ^]^ Compared to flash graphene, LIG benefit from fewer impurities and being a one‐step synthesis‐patterning process. When utilizing lasing to make hybrid nanostructures, the whole process is very rapid, resulting in the formation of LIG‐BDNW films.^[^ ^26^, ^27^ ^]^

Figure 1f shows the Raman spectra of BDNW nanoparticles (spectrum I), and LIG‐BDNW hybrid nanostructures (spectrum II). The characteristic Raman bands for the BDNW nanoparticles were observed at 1154 cm^−1^ (ν_1_‐band), 1340 cm^−1^ (diamond band), 1583 cm^−1^ (G‐band), 2670 cm^−1^ (2D‐band), and 2909 cm^−1^ (D + G band).^[^ ^28^ ^]^ The inset of Figure 1f presents a magnified region of the Raman spectra in the range from 200 to 700 cm^−1^, depicting the B + C band (≈470 cm^−1^) arising due to the boron doping into the diamond lattice. The *ν*_1_‐band at 1154 cm^−1^ originates from the presence of trans‐polyacetylene segments within the diamond, while the G band corresponds to the presence of *sp* ^2^ hybridized carbon atoms in grain boundaries, and the 2D and D + G bands indicate the presence of various kinds of defects.^[^ ^29^ ^]^ The diamond band appears broadened with a slight blue shift (8 cm^−1^) resulting from boron doping and the Fano effect.^[^ ^30^ ^]^

The Raman spectrum of the LIG‐BDNW hybrid structure shown in spectrum II of Figure 1f was analyzed in order to study the defects and variation in graphitization of the LIG due to the incorporation of BDNW. The diamond band for the hybrid structure is found at 1335 cm^−1^ with a full width at half maximum (FWHM) of 35 cm^−1^, implying the high crystallinity of the carbon in the *sp* ^3^ hybridization and indicating the clear presence of diamond in the hybrid structure.^[^ ^8^ ^]^ The LIG‐BDNW sample shows a D‐band at 1350 cm^−1^ and a G‐band at 1581 cm^−1^, attributed to disorder and *sp* ^2^ hybridized carbon, respectively. The 2D band of the hybrid structure fitted with a Lorentzian curve is centered at 2680 cm^−1^ with an FWHM of 96 cm^−1^ and originating from the 2nd order zone boundary phonons, ensuring the graphene quality remained intact. More importantly, the FWHM for the G‐band of the LIG is found to be 45 cm^−1^, whereas for the hybrid structure, it is 43 cm^−1^. The minor improvement in graphitization is due to the presence of BDNW during the photo‐thermal conversion of the Kapton sheet.

### 2.2 Electrical and Field Electron Emission Properties

**Figure 3:**
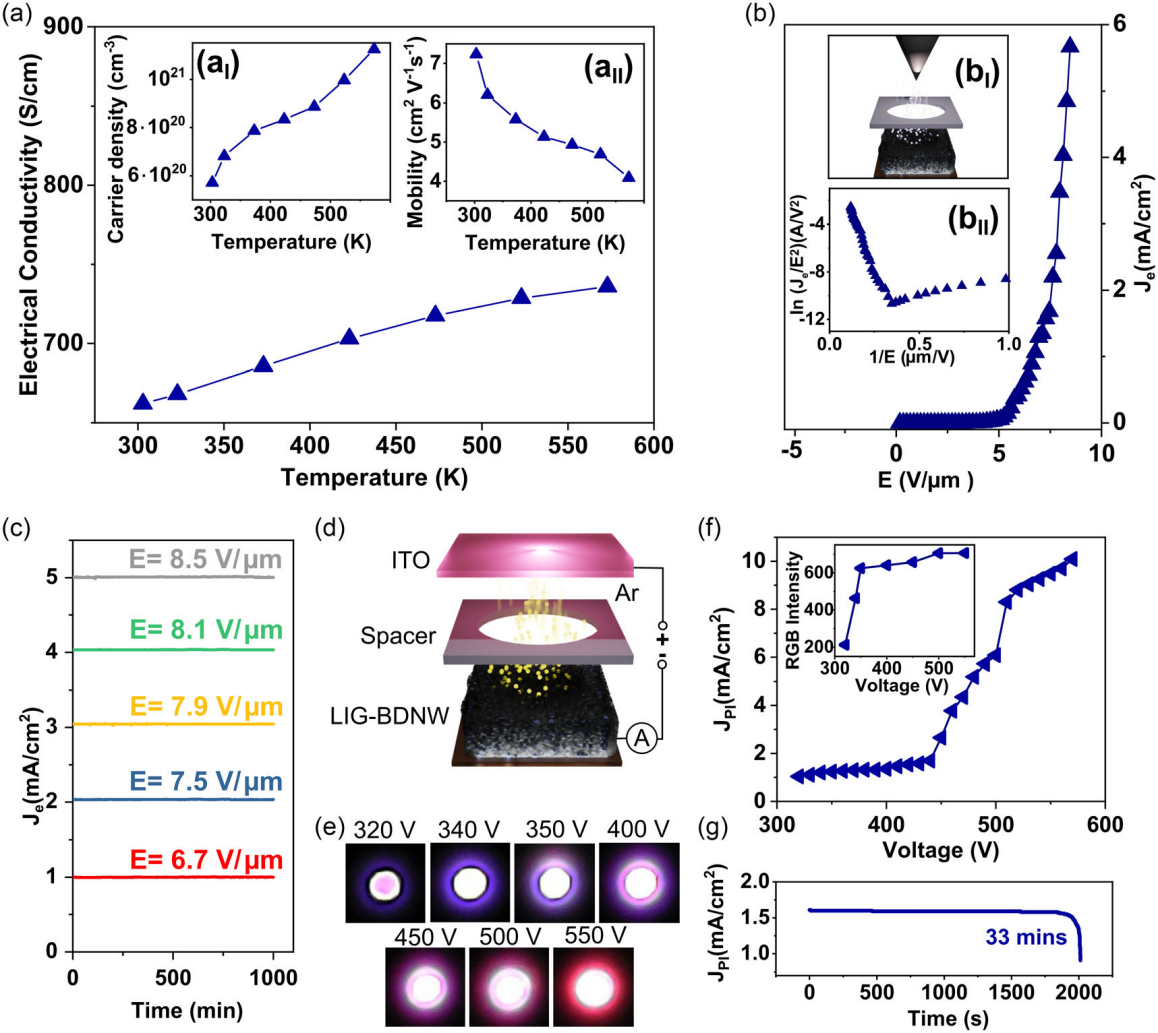
Hall and field effect measurements. a) Temperature‐dependent Hall effect measurements of electrical conductivity with the insets showing the carrier density and mobility for LIG‐BDNW hybrids. b) Field electron emission (FEE) current density (*J*_e_) versus applied field (E) for LIG‐BDNW hybrid was used as the cathode material with the inset showing the schematic of the FEE measurement setup (*b*_I_) and Fowler–Nordheim (F–N) plots corresponding to *J*_e_–*E* characteristic curves (*b*_II_). c) Lifetime stability test, showing *J*_e_ versus time, for LIG‐BDNW hybrids at different applied fields. d) Schematic illustration of the plasma illumination device used in this study consisting of a microplasma cavity configuration, with indium tin oxide (ITO)‐coated glass as the anode. e) Microplasma illumination images at different applied voltages. f) Plasma current density (*J*_PI_) versus applied voltage for LIG‐BDNW hybrid was used as the cathode materials with inset showings the RGB intensity of the same cathode. g) Plasma illumination intensity for LIG‐BDNW at 33 min.

Hall effect measurement in the Van der Pauw configuration was used to investigate the temperature‐dependent electrical conductivity (*σ*), carrier density (*n*_c_), and mobility (*μ*) of the LIG‐BDNW nanohybrids. The obtained results revealed that, at RT, the *σ* value for LIG‐BDNW is 662 S cm^−1^, as depicted in **Figure** **3**a. This shows that incorporating BDNW into the LIG increased the *σ* value as compared the *σ* value of bare LIG (597.6 S cm^−1^)^[^ ^31^ ^]^ and bulk reduced graphene oxide (rGO).^[^ ^32^, ^33^ ^]^ Moreover, Hall measurements were further carried out by increasing the operating temperature from RT to 573 K. As shown in Figure 3a, the *σ* values of the LIG‐BDNW increased monotonically with the temperature.^[^ ^34^, ^35^, ^36^ ^]^

At a maximum temperature of 573 K, the σ value was observed to increase to 735.9 S cm^−1^ for the LIG‐BDNW hybrid. In addition, as depicted in inset a_I_ of Figure 3a, it was observed that at RT, the *n*_c_ value (i.e., concentration of the majority charge carriers) for LIG‐BDNW was found to be 6.1 × 10^20^ cm^−3^. The 2D band in the Raman spectra of graphene is sensitive to free charge carriers and the 2D G^−1^ ratio increases with the charge carrier density.^[^ ^37^ ^]^ So, one can expect this ratio to increase by BDNW incorporation. The carriers’ density increases with the Hall temperature such that they reached 1.26 × 10^21^ cm^−3^ at 573 K.^[^ ^38^, ^39^ ^]^ It is worth mentioning that both electrons and holes are charge carriers in graphene. Upon temperature enhancement, the amount of available thermal energy that can potentially generate charge carriers increases, resulting in an enhanced carrier density. Because of the low doping level of LIG, we can expect this enhancement is mainly related to an increase in the number of electrons. However, because of p‐type doping in BDNW, the charge carriers’ enhancement for LIG‐BDNW hybrid nanostructures probably originates from increasing of the number of both electrons and hole carriers, resulting in a 207% enhancement in the studied temperature range. On the contrary, as depicted in inset a_II_ of Figure 3a, it was observed that at RT, the *μ* value for LIG‐BDNW is 7.2 cm^2^ V^−1^·s following a downward trend with the increase in the Hall temperature and reaches the lowest mobility 4.1 cm^2^ V^−1^·s at 573 K. The decrease in the mobility of LIG‐BDNW with respect to temperature is due to the neutral impurity scattering.^[^ ^40^, ^41^ ^]^

Figure 3b shows the FEE plot of the current density (*J*_e_) as a function of the applied electric field (*E*) for the LIG‐BDNW hybrids. The FEE measurement setup is schematically illustrated as inset *b*_I_ of Figure 3b. The plots of ln(*J*_*e*_/*E* ^2^)v s. 1/*E* (inset *b*_II_ of Figure 3b) show a low turn‐on field (*E*_0_) value of 2.9 V μm^−1^ attained for LIG‐BDNW. To reach a large FEE current density (*J*_e_) value of 3.0 mA cm^−2^, it needs an applied field of *E* = 7.9 V μm^−1^ for LIG‐BDNW (Figure 3b).

Moreover, the field‐enhancement factor (*β*) value can be calculated from the slope of the fitted line using the following equation: *β = *−6.8 × 103 × *φ* ^1.5^/*m*; where m is the slope of the straight line in a high‐field regime. The Fowler–Nordheim (F–N) plots in inset b_II_ of Figure 3b fit well with a straight line, representing that the LIG‐BDNW hybrids obey the F–N rule.^[^ ^42^ ^]^ Generally, the value of *β* is determined from the aspect ratio, tip geometry and screen effect of nanostructured materials. By keeping the *φ* value as 5.0 eV,^[^ ^43^ ^]^ the estimated *β* value for the LIG‐BDNW hybrids is 5480.

The FEE lifetime of the LIG‐BDNW hybrids is a significant factor for field‐emission applications. To investigate the lifetime value of these hybrids, their emission was monitored for different applied voltages—6.7, 7.5, 7.9, 8.1, and 8.5 V μm^−1^—over a period of 1000 min, where the *J*_e_ value increases from 1.0, 2.0, 3.0, 4.0 to 5.0 mA cm^−2^, respectively, with the increase in the applied field (Figure 3c). In all cases, the fluctuations of the *J*_e_ are below 5% and the *J*_e_ values last for more than 1000 min without showing signs of emission current decay, indicating excellent performance in emission stability for these hybrid materials. The obtained FEE results of the LIG‐BDNW hybrids are competitive with the previously reported FEE performance of other carbon hybrid materials, as represented in **Table** **1**.

**Table 1:** Field electron emission characteristics of LIG‐BDNW hybrids compared to other carbon hybrids reported in the literature.

Materials	Turn‐on field [V μm^−1^]	FEE current density [mA cm^−2^]	Field enhancement factor	FEE lifetime
Diamond–graphene hybrid^[^ ^109^ ^]^	3.4	2.57 @ 17.0 V μm^−1^	7.05 × 10^2^	135 h
Carbon nanoflake balls‐Carbon nanotubes^[^ ^110^ ^]^	1.77	–	7,932	50 h
CNTs‐microcrystalline diamond^[^ ^111^ ^]^	2.84	3.59 @ 2.84 V μm^−1^	6,992	100 h
CNTs‐nitrogen‐doped diamond films^[^ ^112^ ^]^	3.58	1.86 @ 6.0 V μm^−1^	2,485	350 min
Single‐walled CNTs grown on single‐walled carbon nanohorn^[^ ^113^ ^]^	12 kV cm^−1^	–	0.79 × 10^5^	–
Carbon nanofilament‐graphene hybrids^[^ ^114^ ^]^	1.34	–	4,932	–
Carbon nanofibres‐UNCD^[^ ^115^ ^]^	2.5	–	–	–
Multilayered graphene‐boron‐doped diamond hybrid nanowalls^[^ ^116^ ^]^	2.4	4.2 @ 4.0 V μm^−1^	4,500	700 min
TiO_2_‐DLC‐Graphene nanocomposites^[^ ^117^ ^]^	5.2	2.95 @ 8.2 V μm^−1^	1,208	–
TiO_2_/Ti nanotubes‐DLC nanorods^[^ ^118^ ^]^	3.0	3.4 @ 8.23 V μm^−1^	–	480 min
Multilayer graphene‐silicon nanowire^[^ ^119^ ^]^	2.42	3.49 @ 3.2 V μm^−1^	1,068	20 h
Graphene‐diamond nanoflakes^[^ ^44^ ^]^	9.3	2.57 @ 24.0 V μm^−1^	2,380	–
hBN‐diamond nanorods^[^ ^120^ ^]^	6.0	4.10 @ 14.0 V μm^−1^	5,870	435 min
Gold‐UNCD hybrids^[^ ^121^ ^]^	2.1	5.30 @ 4.9 V μm^−1^	–	372 min
ZnO‐UNCD nanorods^[^ ^122^ ^]^	2.08	5.5 @ 4.25 V μm^−1^	4,227	140 min
ZnO nanowires‐3D graphene foam^[^ ^123^ ^]^	1.7	–	1,878	2,000 s
CdSe quantum dot‐amorphous CNTs hybrids^[^ ^124^ ^]^	6.74	5.5 μA cm^−2^ @ 15.33 V μm^−1^	–	200 min
ZnO nanowires‐Zn nanoflakes^[^ ^125^ ^]^	0.65	1.4 @ 380 V	18108	180 min
rGO‐CNT films^[^ ^126^ ^]^	2.82	–	3,976	120 min
SnO_2_‐capped silicon nanowires^[^ ^127^ ^]^	3.7	–	4,170	–
Boron‐doped diamond‐carbon nanospine hybrids^[^ ^128^ ^]^	1.3	2.7 @ 2.0 V μm^−1^	6,780	780 min
LIG‐BDNW hybrids^[Present work]^	2.9	3.0 @ 0.79 V μm^−1^	5,480	346 min

### 2.3 Fabrication of a Microplasma Display Device and Microplasma Illumination Properties of LIG‐BDNW‐Based Cathodes

The interesting phenomena in the robustness of the LIG‐BDNW hybrid nanostructures can be very well signified by the fabrication of a microplasma display (PD) device utilizing these hybrids as cathodes because these emitters experience the bombardment of energetic Ar ion in such a device environment, proving their robustness. The schematic of the PD device is illustrated in the inset of Figure 3d. Figure 3e illustrates the μPI images of LIG‐BDNW hybrids, with the voltage sweeping from 300 to 550 V. It was observed from Figure 3e that the μPI intensity increases with the applied voltage, resulting in a highly intense plasma being observed at an applied voltage of 550 V.^[^ ^44^, ^45^ ^]^ The local electric field across the cathode microstructure increases with the applied voltage, resulting in enhanced μPI. The breakdown voltage (*V*_bk_) for the LIG‐BDNW hybrid was 320 V. This supports the previous discussion that enhanced electrical conductivity in LIG‐BDNW hybrids aids the development of a stronger electrical field across cathode materials. Strong electric fields at the edges of both LIGs and BDNWs enhances the electron energy level, resulting in the formation of hot carriers.^[^ ^46^, ^47^ ^]^ It is worth mentioning that defects in the LIG structure significantly enhance the electron emission.^[^ ^48^ ^]^ Inset of Figure 3f depicts the intensities of red, green, and blue (RGB) versus applied voltage for LIG‐BDNW; these values were calculated from the plasma images as shown in the inset of Figure 3e. The curve clearly displays a higher μPI intensity for the hybrid nanostructures. Furthermore, it is evident that the RGB intensity increases with the applied voltage in the range of 300–550 V. FEE and μPI properties of carbon materials originate from the energy level of electrons, which is affected by various factors such as electric field (i.e., applied voltage for a predefined device configuration) and its distribution throughout the hybrid nanostructure, temperature, and intrinsic features of nanomaterials that are dictated by their chemical composition, impurities, doping level, and defects. Higher electrical conductivity of cathode materials results in less Ohmic drop and the development of higher electrical fields across the electrodes. In addition, sharp‐edged nanostructures, such as graphene nanoplatelets and faceted BDNW, are prone to developing stronger local electric fields.^[^ ^49^, ^50^, ^51^ ^]^ Accordingly, one can expect enhanced electron emission sites from the numerous edges of graphene nanoplatelets and BDNWs, as previously observed in the SEM images.

Figure 3f depicts the variation of the μPI current density (*J*_PI_) versus applied voltage; at breakdown voltages, that is, 320 V for LIG‐BDNW‐based cathodes, the *J*_PI_ values were measured to be 9.48 mA cm^−2^ at an applied voltage of 550 V. The high value of *J*_PI_ for LIG‐BDNW‐based cathodes is because of the higher applied voltages, resulting in a stronger electric field and enhanced emission. As discussed earlier, this is probably because of the enhanced electrical properties of hybrid nanostructures and faceted structure of BDNW corresponding to an increased number of edges with a stronger local field.

Regardless of the breakdown voltage, current density and plasma density, the long‐term stability of cathode materials under a harsh plasma environment is crucially important for the development of PD devices. Figure 3g shows the lifetime stability curve for the LIG‐BDNW hybrid cathodes, for which *J*_PI_ versus time is depicted in a harsh plasma environment. This figure shows that the lifetime stability for LIG‐BDNW cathodes is 33 min. The superior μPI characteristics of LIG‐BDNW hybrids are comparable with the μPI characteristics of other carbon hybrid‐based cathodes utilized in a PD device (**Table** **2**). In fact, the harsh conditions during electron emission and μPI, and enhanced temperature, can result in the rearrangement of atoms, de‐doping (i.e., release of boron), and even partial oxidation of carbon atoms in the LIG‐BDNW hybrid cathodes, resulting in diminished performance of these structures over time.

**Table 2:** Plasma illumination characteristics of LIG‐BDNW hybrids compared to other carbon hybrids‐based microplasma cathodic devices reported in the literature.

Materials	Breakdown voltage [V]	Plasma current density [mA cm^−2^]	PI lifetime
Multilayered graphene‐boron‐doped diamond hybrid nanowalls^[^ ^116^ ^]^	330	6.0 @ 510 V	358 min
Graphene‐diamond nanoflakes^[^ ^44^ ^]^	380	3.8 @ 570 V	21 min
hBN‐diamond nanorods^[^ ^120^ ^]^	350	1.04 @ 540 V	29 min
Gold‐UNCD hybrids^[^ ^121^ ^]^	370	3.0 @ 520 V	7.12 h
ZnO‐UNCD nanorods^[^ ^122^ ^]^	160	3.97 @ 300 V	107 min
Boron‐doped diamond‐carbon nanospine hybrids^[^ ^128^ ^]^	270	16.2 @ 500 V	545 min
Graphite‐diamond nanohybrid films^[^ ^129^ ^]^	320	2.15 @ 510 V	491 min
Few‐layer graphene‐diamond nanorods^[^ ^43^ ^]^	540	14.80 @ 900 V	20 min
Nitrogen‐doped diamond films‐CNTs^[^ ^112^ ^]^	360	1.88 @ 550 V	218 min
LIG‐nanodiamond particles^[^ ^31^ ^]^	330	5.8 @ 550 V	17 min
LIG^[^ ^31^ ^]^	310	7.6 @ 550 V	≈12 min
LIG‐BDNW hybrids^[Present work]^	320	9.48 @ 550 V	33 min

### 2.4 Advanced Characterization of Hybrid Nanostructures

**Figure 4:**
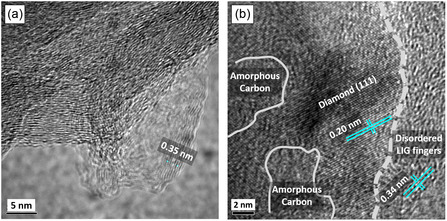
TEM results for nanostructures. a) HRTEM image of LIG, interlayer spacing ≈0.35 nm, aligns with the (002) planes of graphitized materials. Scale bar 5 nm. b) HRTEM image of LIG‐BDNW composite showing that the diamond (111) plane is surrounded by disordered graphitic carbon and amorphous carbon, respectively. Scale bar 2 nm.

The HRTEM image shown in **Figure** **4**a reveals the interlayer spacing of LIG is roughly ≈0.35 nm, corresponding to the (002) plane of graphite. This specific interlayer spacing confirms the existence of a few‐layer graphene structure embedded within the LIG. Note that characteristic wrinkles are also visible in the graphitized PI film, induced by compressive strain during laser irradiation.^[^ ^52^ ^]^ In the HRTEM image (Figure 4b) of the LIG‐BDNW composite, we can identify diamond lattice planes (111). The distance between these planes measures about 0.20 nm, which matches the spacing of diamond (111) planes in nanodiamond crystals.^[^ ^53^ ^]^ Also, we noticed evidence of amorphous carbon and disordered fringes of LIG (*d *≈ 0.34 nm) along the grain boundaries of the diamond crystallites. The HRTEM results further confirm the successful decoration of BDNW particles onto the LIG network.

**Figure 5:**
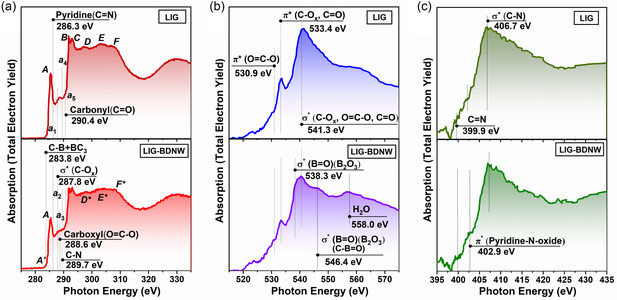
NEXAFS spectroscopy results. a) C1*s* X‐ray absorption spectrum of LIG and LIG‐BDNW hybrids. b) O1*s* X‐ray absorption spectrum of LIG and LIG‐BDNW hybrids. c) N1*s* X‐ray absorption spectrum of LIG and LIG‐BDNW hybrids.

**Figure 6:**
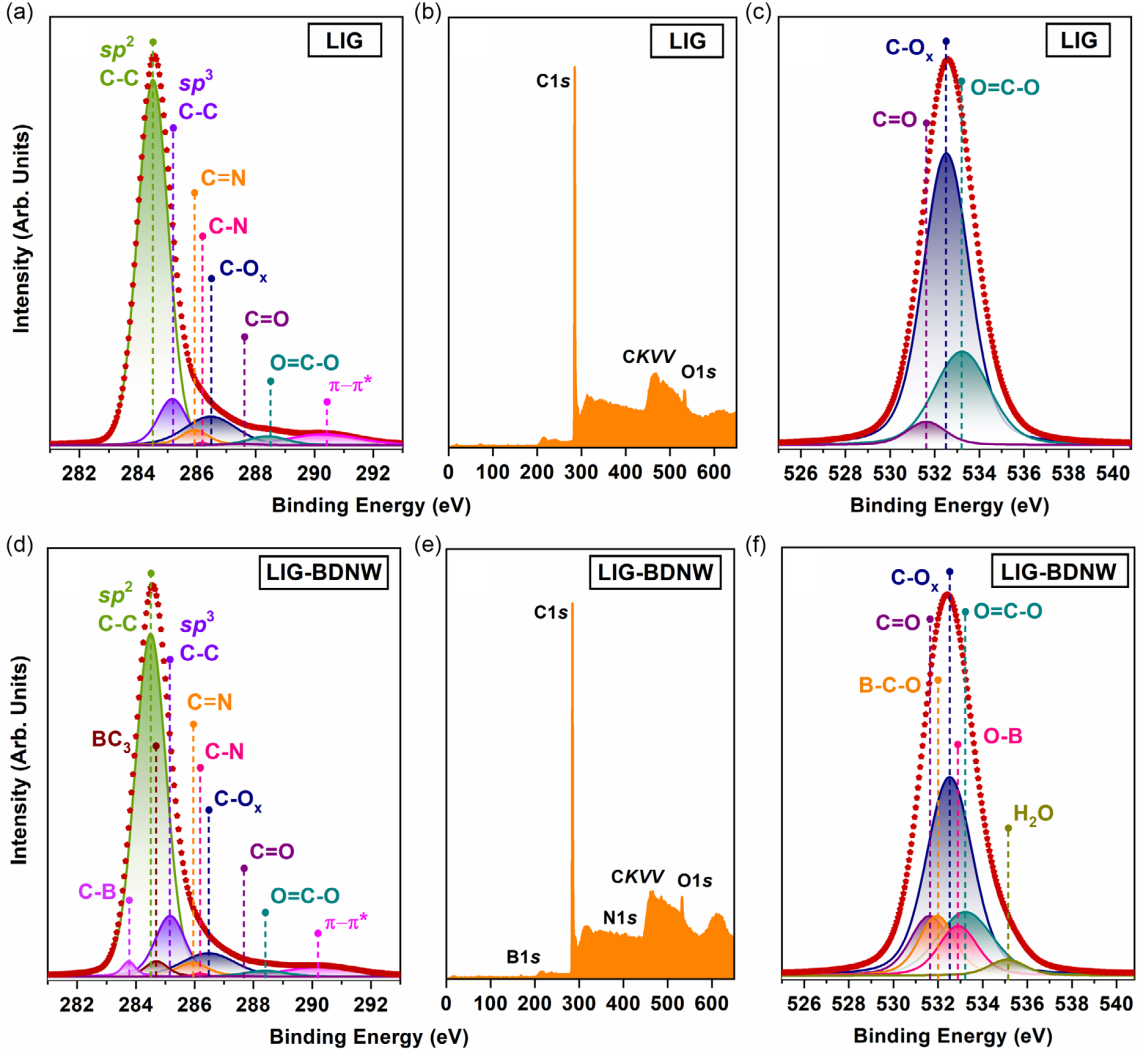
High‐resolution X‐ray photoelectron spectra: a) C1*s*, c) O1*s*, b) survey spectra for LIG‐P, d) C1*s*, f) O1*s*, and e) survey spectra for LIG‐BDNW.

The results obtained by characterizing both LIG and LIG‐BDNW samples using microscopic and Raman spectroscopy methods were also confirmed by near‐edge X‐ray absorption fine structure (NEXAFS) spectroscopy. **Figure** **5** shows the C1*s* (Figure 5a), O1*s* (Figure 5b) and N1*s* (Figure 5c) X‐ray absorption spectra of the LIG and LIG‐BDNW. The C1*s* X‐ray absorption spectrum of the LIG perfectly reproduces all the main features of the spectra of highly oriented pyrolytic graphite (HOPG) and single‐layer graphene (SLG) that we acquired earlier.^[^ ^54^, ^55^ ^]^ First, this applies to absorption peaks (resonances) *A* and *B*–*C*. These peaks are associated with dipole‐allowed transitions of the 1*s* electrons of the carbon atom into free states of *π*‐ and *σ*‐symmetry of the conduction band of a SLG and graphite crystal, which are formed from the *π*2*p*_z_‐ and *σ*2*p*_x,y_‐states of carbon atoms oriented, respectively, perpendicularly and parallel to the graphene plane.^[^ ^56^ ^]^ Moreover, like HOPG and SLG, broad *D*–*F* absorption bands are observed in the LIG spectrum, which reflect electronic transitions to free *σ* states of the conduction band of graphite and graphene associated with the interaction of carbon hexagons in the graphene layer.^[^ ^57^ ^]^ In addition, in the case of the LIG, almost the same difference in the position of the peaks *A*–*B* and *A*–*C* is observed, Δ*E*_*A–B*_ = 6.4 eV and Δ*E*_*A–C*_ = 7.55 eV, as in the case of HOPG and graphene (Δ*E*_*A–B*_ = 6.4 eV, Δ*E*_*A–C*_ = 7.4 eV). It is also important that the FWHM of peak *A* has a value close to those for HOPG (FWHM = 1.15 eV) and graphene (FWHM = 1.55 eV), namely FWHM = 1.2 eV. It is necessary to emphasize that the LIG spectrum does not have a feature in the form of a step at *hv* = 283.7 eV.^[^ ^58^ ^]^ This indicates that there is no chemical bonding between the LIG and the substrate on which it is deposited. In addition, the absence of this step indicates a few‐layer graphene structure in the LIG, which is also confirmed by our HRTEM data.^[^ ^59^ ^]^ All the aforementioned facts clearly indicate the high quality of the resulting LIG, the carbon atoms of which have a predominant *sp* ^2^ type of hybridization. It seems logical to associate the observed broadening of the *π*‐band A with the splitting of the *π*2*p*_z_ conduction subband because of a slight corrugation of the flat single‐layers’ graphene and a decrease in their symmetry in LIG due to a small number of defects in graphene layers,^[^ ^60^ ^]^ as well as the chemical bonding of carbon atoms with oxygen and nitrogen atoms. The presence of such chemical bonding is also confirmed by the appearance in the LIG spectrum of five weak additional features—*a*_1_, *a*_2_, *a*_3_, *a*_4_, and *a*_5_—between the *π* and *σ* resonances. The presence of small amounts of chemically bound oxygen and nitrogen in the LIG is also confirmed by the O1*s* (Figure 5b) and N1*s* (Figure 5c) X‐ray absorption spectra of the LIG and our photoemission measurements (**Figure** **6**). Features *a*_1_ (*hv* = 286.5 eV) and *a*_4_ (*hv* = 289.7 eV) are due to transitions of C1*s* electrons to free 2*p* states of carbon atoms in areas of nitrogen‐doped graphene, with the formation of pyridine C=N and C—N bonding, respectively.^[^ ^61^ ^]^ The features *a*_2_ (*hv* = 287.8 eV), *a*_3_ (*hv* = 288.7 eV), and *a*_5_ (*hv* = 290.4 eV) are due to the transitions of C1*s* electrons to free 2*p* states of carbon atoms on the surface areas of the LIG, oxidized during its synthesis, with the formation of (C—Ox), carboxyl (O=C—O) and carbonyl (C=O) bonds, respectively.^[^ ^62^, ^63^, ^64^ ^]^

In the O1*s* X‐ray absorption spectrum of the LIG (Figure 5b), three main features are clearly visible at *hv* = 530.3 eV, *hv* = 533.4 eV, and *hv* = 541.3 eV, which are also caused by transitions of O1*s* electrons to free 2*p* state oxygen atoms on the surface areas of the LIG, oxidized during its synthesis, with the formation of π*(O=C—O), π*(C—Ox, C=O), and σ*(C—Ox, O=C—O, C=O) bonds, respectively.^[^ ^65^, ^66^ ^]^

In the N1*s* X‐ray absorption spectrum of LIG (Figure 5c), a main broad absorption band is observed, centered at *hv* = 406.7 eV. This band corresponds to transitions from N1*s* core levels to unoccupied σ*(C—N) orbitals.^[^ ^67^ ^]^ Further, two low‐intensity features with *hv* = 399.9 eV and *hv* = 402.9 eV are distinguishable in the spectrum, which are caused by transitions of N1*s* electrons to free 2*p* states of nitrogen atoms in areas of nitrogen‐doped graphene, with the formation of π*(C=N)^[^ ^74^ ^]^ and π*(pyridine‐N‐oxide) bonds, respectively.^[^ ^68^ ^]^

Unlike the LIG, the C1*s* X‐ray absorption spectrum of the LIG‐BDNW (Figure 5a) is a superposition of the C1*s* X‐ray absorption spectra of graphene and nanodiamonds. This is indicated by the fact that, on the one hand, the C1*s* spectrum of LIG‐BDNW reproduces the main features of the spectra of HOPG and graphene, namely, *π* and *σ* resonances (peaks *A* and *B*‐*C*), and the distance between them is preserved (Δ*E*_*A*–*B*_ = 6.4 eV and Δ*E*_*A*–*C*_ = 7.55 eV). On the other hand, the LIG‐BDNW spectrum has a shape in the spectral range of *hv* = 296 ÷ 315 eV with D*‐F* peaks, which is more typical for the C1*s* X‐ray absorption spectrum of nanodiamonds than for graphene.^[^ ^69^, ^70^ ^]^ In addition, peak *B* has a lower intensity than peak *C* in the LIG‐BDNW C1*s* spectrum, which is also characteristic of the spectrum of nanodiamonds. It should be noted that peak *A* in the C1*s* absorption spectrum of the LIG‐BDNW is slightly broadened (FWHM = 1.3 eV) compared to the C1*s* absorption spectrum of the LIG, which is a consequence of the presence of disordered fringes of LIG along the grain boundaries of the diamond crystallites (see Figure 4b). Thus, the analysis of the C1*s* X‐ray absorption spectrum of the LIG‐BDNW first confirms the conclusion made previously in the analysis of the HRTEM images that BDNW particles decorated successfully onto the LIG network, while the interface of BDNW particles‐LIGs simulates inorganic Janus nanostructures. Similar to the LIG, the C1*s* X‐ray absorption spectrum of the LIG‐BDNW also exhibits five weak additional features—*a*_1_ (*hv* = 286.5 eV), *a*_2_ (*hv* = 287.8 eV), *a*_3_ (*hv* = 288.7 eV), *a*_4_ (*hv* = 289.7 eV), and *a*_5_ (*hv* = 290.4 eV)—between the *π*‐ and *σ*‐resonances, which are associated with the formation of pyridine C=N, (C—Ox), carboxyl (O=C—O), C—N, and carbonyl (C=O) bonds, respectively. Unlike LIG, a new feature – *A*′ (*hv* = 283.8 eV)—is observed in the C1*s* X‐ray absorption spectrum of the LIG‐BDNW, which is caused by transitions of C1s electrons to free 2*p* states of boron atoms in the boron‐doped areas of BDNW, with the formation of C—B^[^ ^71^ ^]^ and BC3 bonds,^[^ ^70^ ^]^ respectively.

In the O1*s* X‐ray absorption spectrum of the LIG‐BDNW, as well as in the LIG spectrum (Figure 5b), three features are clearly visible with *hv* = 530.3 eV, *hv* = 533.4 eV, and *hv* = 541.3 eV, which are caused by O1*s*‐electron transitions into free 2*p* states of oxygen atoms on the surface areas of the LIG and BDNW, oxidized during their synthesis, with the formation of π*(O=C—O), π*(C‐Ox, C=O), and σ*(C—Ox, O=C—O, C=O) bonds, respectively. In addition to this, two other features at *hv* = 538.1 eV and *hv* = 546.4 eV are also clearly distinguishable, which correspond to transitions of O1*s* electrons to free 2*p* states of boron atoms on boron‐doped areas of the BDNW surface with the formation of σ*(C—B=O) bonds.^[^ ^72^ ^]^ Moreover, there is a broadened peak in the spectrum with *hv* = 558 eV, which is characteristic of H_2_O. It can be assumed that this is due to the high affinity for water adsorption of LIG‐BDNW.

The N1*s* X‐ray absorption spectrum of the LIG‐BDNW is identical to the N1*s* X‐ray absorption spectrum of the LIG (Figure 5c). It also exhibits a main broad peak, centered at *hv* = 407 eV. This peak corresponds to transitions from N1*s* core‐levels to unoccupied σ*(C—N) orbitals.^[^ ^67^ ^]^ Additionally, two low‐intensity features with *hv* = 399.9 eV and *hv* = 402.9 eV are distinguishable in the spectrum, which are caused by transitions of N1*s* electrons to free 2*p* states of nitrogen atoms in areas of nitrogen‐doped graphene, with the formation of π*(C=N)^[^ ^61^ ^]^ and π*(pyridine‐N‐oxide) bonds, respectively.^[^ ^68^ ^]^

In order to detail the information on the atomic and electronic structure obtained by NEXAFS spectroscopy of the nanostructures under investigation, we also applied X‐ray photoelectron spectroscopy. It should be especially emphasized that NEXAFS spectroscopy and XPS with excitation by synchrotron radiation were used for the first time in this work to investigate LIG and LIG‐BDNW. Figure 6 demonstrates the high‐resolution C1*s* (Figure 6a,d), O1*s* (Figure 6c,f), and survey (Figure 6b,e) photoelectron spectra of the LIG and LIG‐BDNW samples. All spectra were measured with *hv* = 730 eV. This excitation energy was chosen in such a way that, on the one hand, it allowed us to select the probing depth of the nanostructures as efficiently as possible, and on the other hand, made it possible to obtain maximum sensitivity to the features of the electronic structure of the nanomaterials under investigation.

In the survey X‐ray photoelectron spectrum of LIG, signals from carbon and oxygen atoms can be easily observed as shown in Figure 6b. The high‐resolution C1*s* photoelectron spectrum of LIG has a complex structure (Figure 6a). It is well described by a fitting with eight components, one of which has an asymmetric shape while the other seven components are symmetrical. The main component (plotted in green) in the spectrum is located at a binding energy of 284.5 eV (*BE* = 284.5 eV) and has an asymmetrical shape. It is described using the Doniach–Sunjic function, which is well‐known for HOPG and graphene.^[^ ^73^, ^74^ ^]^ Thus, it can be identified with the C=C phase with *sp* ^2^ hybridization of the valence electron states of the carbon atoms. The relative intensity of this component, estimated by comparing the areas under the peaks, is 71%. This observation confirms the conclusion made above when analyzing the NEXAFS spectrum that the fabricated LIG has a very good, high‐quality graphene mesh. This is also confirmed by the presence in the spectrum of a *π*–π* satellite with *BE* = 290.3 eV (plotted in magenta).^[^ ^75^ ^]^ Another conclusion drawn above about the presence of an insignificant number of defects in the LIG is confirmed by the presence in the sample of a small number of carbon atoms with *sp* ^3^ hybridization of the valence electron states (8.8%). This demonstrates the presence of a component with *BE* = 285.2 eV (plotted in violet).^[^ ^76^ ^]^ The appearance of the remaining five components should be considered as a consequence of chemical bonding between carbon, nitrogen and oxygen atoms. This interaction leads to the formation of oxygen‐containing and nitrogen‐containing functional groups and is accompanied by a transfer of charge from carbon atoms to nitrogen and oxygen atoms due to their higher electronegativity. The presence of nitrogen‐containing functional groups is confirmed by the presence of components with *BE* = 285.9 eV (plotted in orange) and *BE* = 286.2 eV (plotted in pink). Therefore, these components can be identified with C=N^[^ ^77^ ^]^ and C—N types of bonds in graphene,^[^ ^78^ ^]^ and their relative content in LIG is 3.1% and 0.2%, respectively. The other three components have higher binding energies, namely *BE* = 286.5 eV (plotted in navy), *BE* = 287.5 eV (plotted in purple), and *BE* = 288.4 eV (plotted in cyan). The presence of these components in the C1*s* photoelectron spectrum confirms the presence of oxygen‐containing groups in the LIG, such as C—O_x_ with a relative content of 9.5%, C=O with a relative content of 0.3%, and O=C—O with a relative content of 2.3%, respectively.^[^ ^77^ ^]^ The presence of the same oxygen‐containing groups in the LIG is also confirmed by the presence of three components in the O1*s* photoelectron spectrum of the LIG sample (Figure 6c). These three components have binding energies *BE* = 532.5 eV (plotted in navy), *BE* = 531.6 eV (plotted in purple), and *BE* = 533.2 eV (plotted in cyan). Therefore, they can be identified with C—O_x_, C=O (carbonyl), and O=C—O (carboxyl) types of bonds, and their relative content in the LIG is 69.2%, 4.8%, and 26%, respectively.^[^ ^74^ ^]^ Thus, we can state that the main fraction of oxygen in the LIG is a consequence of surface contamination.

Thus, the conclusions drawn from the analysis of the X‐ray photoelectron spectra of the LIG are in full agreement with the conclusions formulated from the analysis of their X‐ray absorption spectra. The same is true for the LIG‐BDNW. In the survey X‐ray photoelectron spectrum of the LIG‐BDNW, signals from carbon, nitrogen, oxygen, and boron atoms can be easily seen (Figure 6e). The high‐resolution C1*s* photoelectron spectrum of the LIG‐BDNW also has a complex structure (Figure 6d). Compared to the LIG, it is well described by a fitting with 10 components, one of which has an asymmetric shape and the other nine components being symmetrical. The main component (plotted in green) in the spectrum is located at a binding energy of 284.5 eV (*BE* = 284.5 eV) and has an asymmetrical shape. It is described using the Doniach–Sunjic function, which is well known for HOPG and graphene, as well.^[^ ^73^, ^74^ ^]^ Thus, it can be identified with the C=C phase with *sp* ^2^ hybridization of the valence electron states of the carbon atoms. The relative intensity of this component, estimated by comparing the areas under the peaks, is 66.5%. This value is 4.5% less than for the LIG. A *π*–π* satellite with *BE* = 290.3 eV (plotted in magenta) is also observed in the C1*s* photoelectron spectrum of the LIG‐BDNW.^[^ ^75^ ^]^ The relative intensity of this component is also slightly lower than in the C1*s* photoelectron spectrum of the LIG, namely 4.3%. At the same time, the relative intensity of components with *BE* = 285.2 eV (plotted in violet), which corresponds to carbon atoms with *sp* ^3^ hybridization of the valence electron states,^[^ ^76^ ^]^ is 3.2% smaller than in the LIG, namely 12%. These observations are in full agreement with the structure of the LIG‐BDNW, which is graphene with the addition of BDNWs. The appearance of the remaining seven components should be considered as a consequence of chemical bonding between atoms of carbon, nitrogen, oxygen and boron. Moreover, five of these components are located far on the side of high binding energies from the main peak, like the C1*s* photoelectron spectrum of the LIG. From the other two components, one component is located on the lower binding energy side of the main peak, and the other component is located on the higher binding energy side. These five high‐energy components are a consequence of the formation of oxygen‐containing and nitrogen‐containing functional groups and are accompanied by a transfer of charge from carbon atoms to nitrogen and oxygen atoms due to their greater electronegativity. Components with *BE* = 285.9 eV (plotted in orange) and *BE* = 286.2 eV (plotted in pink) can be identified with C=N^[^ ^77^ ^]^ and C—N types of bonds in graphene,^[^ ^78^ ^]^ and their relative content in the LIG‐BDNW is 3.1% and 0.3%, respectively. This is like the LIG. The other three high‐energy components have higher binding energies, namely *BE* = 286.5 eV (plotted in navy), *BE* = 287.5 eV (plotted in purple), and *BE* = 288.4 eV (plotted in cyan). The presence of these components in the C1*s* photoelectron spectrum also confirms the presence of oxygen‐containing groups in the LIG‐BDNW, such as C—O_*x*_ with a relative content of 8%, C=O with a relative content of 0.1%, and O=C—O with a relative content of 1.6%.^[^ ^77^ ^]^ Thus, the content of oxygen‐containing groups in the LIG‐BDNW is slightly lower than in the LIG. The low‐energy component is a consequence of the formation of boron‐containing functional groups and is accompanied by a transfer of charge from boron atoms to carbon atoms due to their lower electronegativity. The component with *BE* = 283.8 eV (plotted in lilac) can be identified with C—B nanodiamonds,^[^ ^70^, ^78^ ^]^ and its relative content in LIG‐BDNW is 1.6%. The component with *BE* = 284.7 eV (plotted in brown) can be identified with BC_3_ nanodiamonds,^[^ ^79^ ^]^ and their relative content in the LIG‐BDNW is 2.4%.

The presence of the same oxygen‐containing groups in the LIG‐BDNW is also confirmed by the presence of three of the six components in the O1*s* photoelectron spectrum (Figure 6f). These three components have binding energies of *BE* = 532.5 eV (plotted in navy), *BE* = 531.6 eV (plotted in purple), and *BE* = 533.2 eV (plotted in cyan). Therefore, they can be identified with C—O_x_, C=O (carbonyl), and O=C—O (carboxyl) types of bonds, and their relative content in the LIG‐BDNW is 46.4%, 11.9%, and 17.5%, respectively.^[^ ^74^ ^]^ Thus, the main fraction of oxygen in the LIG‐BDNW is also a consequence of surface contamination. It should be especially emphasized that there are three additional components in the O1*s* photoelectron spectrum of the LIG‐BDNW. The component with *BE* = 532.2 eV (plotted in orange) can be identified with the B—C—O bond types in BDNW.^[^ ^80^ ^]^ Its relative content in the LIG‐BDNW is 11.3%. The component with *BE* = 532.9 eV (plotted in pink) can be identified with O—B bond types in BDNW.^[^ ^79^ ^]^ Its relative content in the LIG‐BDNW is 9.9%. According to Massahi et al. this component can be linked to the formation of a phase such as B_2_O_3_.^[^ ^81^ ^]^ The component with *BE* = 532.9 eV (plotted in dark yellow) can be identified with the H_2_O bond types in the LIG‐BDNW.^[^ ^74^ ^]^ Its relative content in the LIG‐BDNW is 3%. Thus, it is worth emphasizing once again that the conclusions drawn from the analysis of the X‐ray photoelectron spectra of the LIG‐BDNW are in full agreement with the conclusions formulated after the analysis of the X‐ray absorption spectra.

Numerous attempts have been made to improve the electronic properties of graphene, and one of the most convenient and popular strategies is the incorporation of dopants into the graphene nanostructure. Additionally, incorporating nitrogen and boron into graphene sheets has been explored as a feasible strategy to modify their electronic characteristics due to the fact that they have atomic sizes comparable to that of the carbon atom.^[^ ^82^, ^83^, ^84^ ^]^ In addition, these dopants can be used to engineer the bandgap and enhance the catalytic activity of graphene.

Nitrogen is considered an excellent choice for the chemical doping of graphene materials because it has a comparable atomic size and contains five valence electrons available to form strong valence bonds with carbon atoms.^[^ ^85^ ^]^ Nitrogen doping has proven to be an effective strategy for modifying the properties of graphene and preparing it for various potential applications. Three common C—N bonding configurations are normally obtained when doping nitrogen into the graphene lattice: pyridinic N, pyrrolic N, and graphitic N. However, LIG, being fabricated from the decomposition of the Kapton sheet, inherits nitrogen as *sp* ^2^ hybridized carbon.^[^ ^86^ ^]^ Therefore, pristine LIG shows good electrical conductivity and low breakdown voltage.

Furthermore, boron‐doped diamond has found wide employment in various electrical applications.^[^ ^87^ ^]^ The doping of boron into diamond lattice induces electron deficiency corresponding to p‐type semiconductivity, while retaining its original *sp* ^3^ hybridization. Since the electronegativity of boron is smaller than that of carbon, doping with boron can lead to a clear differentiation of electron densities. This leads to improved electrical properties in the case of boron‐doped diamond (BDD).^[^ ^88^ ^]^

The CO_2_ laser is known to have considerably lower photon energy than the bandgap of diamonds (≈5.5 eV). Hence, the laser irradiation of the nanodiamond dispersion photothermally degrades the grain boundaries of the diamond, thus converting *sp* ^3^ carbon into graphitized and/or amorphous carbon.^[^ ^12^, ^89^ ^]^ Since the diamond is dispersed on the PI films before graphene synthesis, the photothermal annealing process, which produces the graphene and degrades the BDNW, ensures the bonding of the two differently hybridized carbon materials, resulting in a LIG‐BDNW hybrid. The improved electrical conductivity, along with the robust nature of the diamond, makes LIG‐BDNW a highly stable and suitable cathode material for microplasma devices.

### 2.5 DFT Analysis: Theoretical Insights into the Electronic Properties of LIG‐BDNW

**Figure 7:**
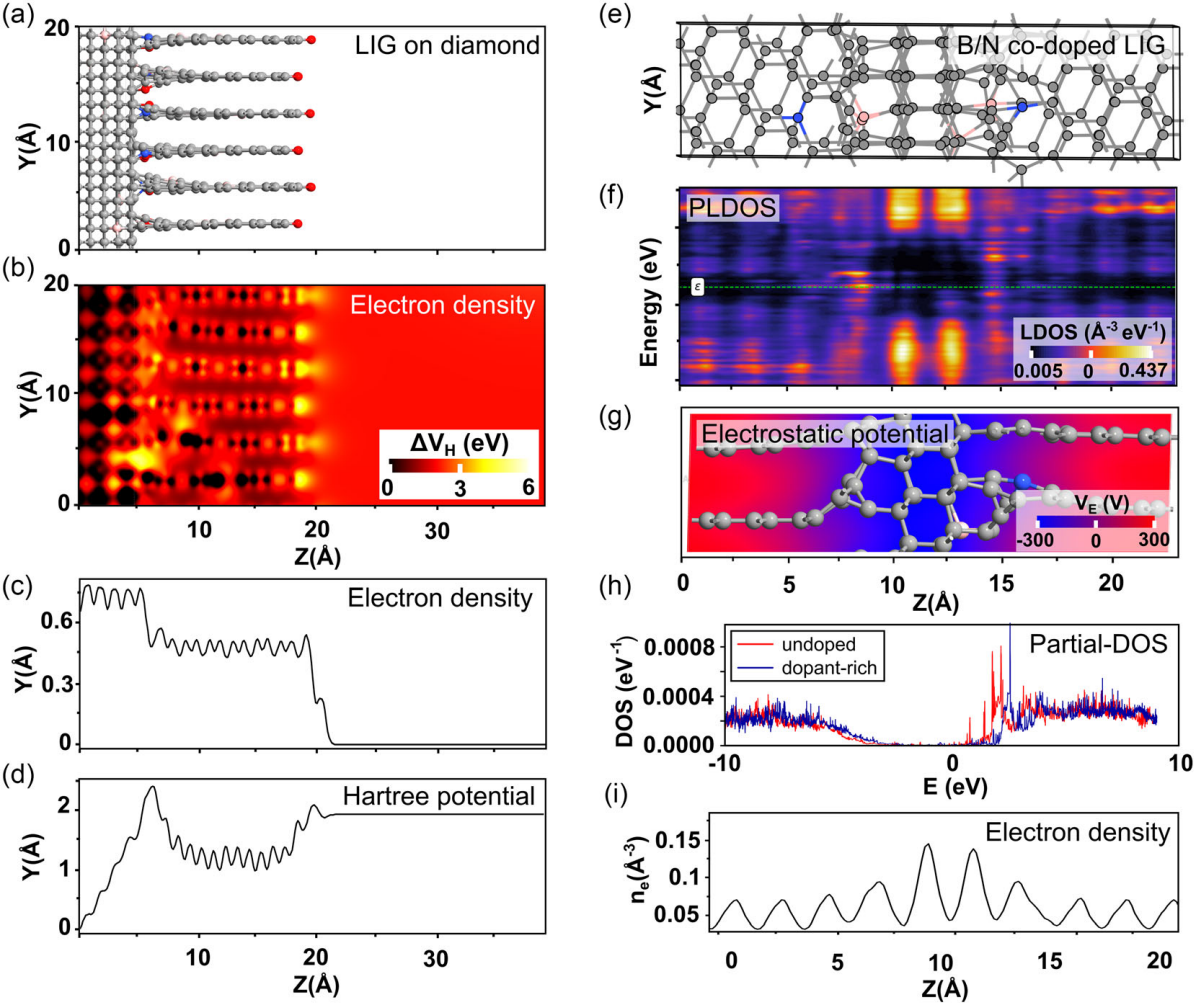
Ab‐initio simulation of LIG: a) Surface LIG‐BCNW slab structure grown on a diamond base with color‐coded atoms: gray – carbon, red – oxygen, blue – nitrogen, pink – boron. b) electron density of the LIG structure represented as a color plot. c) electron density of the LIG structure, represented as the average density plotted in the Z‐axis. d) Hartree potential is represented as the average potential plotted on the Z‐axis. e) interface LIG‐BCNW slab with Y and Z plot (top view). f) PLDOS of the bulk‐doped LIG structure. g) electrostatic potential map plotted on the X‐Y plane of the boron/nitrogen‐doped LIG. h) PDOS of a carbon atom in the LIG structure, with and without boron doping i) electron density of the boron/nitrogen‐doped LIG represented in a 2D plot.

Two computational slab configurations were established to reveal interfacial phenomena and surface characteristics of BDNW‐LIG. The first configuration examined LIG‐BDNW surface performance through a mixed interface atomistic model (**Figure** **7**a–f), comprising diamond and graphene surfaces co‐doped explicitly with boron and nitrogen. The *sp* ^3^‐rich sections simulated diamond cores of particles, while graphenic and *sp* ^3^–*sp* ^2^ hybridized interfaces mimicked the interface between the diamond, BCNW, and LIG. Gray atoms represent carbon, blue nitrogen, pink boron, and red oxygen. The atomic composition of the surfaces and their terminations were designed in accordance with XPS and NEXAFS measurements.

Figure 7b illustrates the electron density of the emulated LIG‐BDNW surfaces. High density is depicted in yellow. The edges of graphene in the LIG‐BDNW surfaces terminated with oxygen and areas doped with nitrogen show high electron density, indicating enhanced electron emission from these sections. Low electron density is primarily observed in diamond cores and around boron atoms. Furthermore, Figure 7c plots the average electron density, which is higher for *sp* ^3^‐hybridized carbon and lower for *sp* ^2^‐rich regions.

The Hartree potential (Figure 7d) plotted along the *Z*‐axis revealed that the graphene‐rich sections exhibited regular potential fluctuations.

Figure 7e displays the BDNW‐LIG interfacial model used for the projected local DOS (PLDOS) calculation, shown from the top (*YZ*‐axis). The calculated PLDOS is plotted in Figure 7f, demonstrating changing electronic properties throughout the structure. In the middle, where *sp* ^3^‐rich core diamond is present, a charge gap in the DOS was detected. As the dopant concentration increases in other sections of the structure, the gap closes (edges of *sp* ^3^). The *sp* ^2^‐rich graphene shows a low bandgap, primarily due to nitrogen doping, as also reported previously in simulations by Qu and coworkers.^[^ ^90^ ^]^ Formation energy calculations indicated that nitrogen is more effectively incorporated into nanocarbons than boron atoms. Nitrogen doping enhances the electron emission by lowering the work function and increasing the emission current, due to an upshift in the Fermi level caused by the local state delivered here by BCNWs. Thereafter, boron doping has a minimal impact on the field‐emission properties of nanocarbons.

Figure 7g presents the LIG‐oriented model from the side (*XZ*‐axis) in an electrostatic density plot. As in the previous model, *sp* ^3^ exhibits a more negative density than *sp* ^2^‐rich sections. The interfaces between the two forms of carbon, representing diamond cores and graphene from BDNW and LIG, typically show the lowest potential.^[^ ^91^ ^]^ The partial density of states (PDOS) for carbon atoms in the LIG structures is plotted in Figure 7h, showing the gap difference between undoped LIG and LIG doped with BDNW.^[^ ^92^ ^]^ Previous research by Nath et al.^[^ ^93^ ^]^ reported similar partial density of states (DOS) characteristics, demonstrating that the bandgap position in graphene sheets is influenced by the type of dopant introduced. Furthermore, the study conducted by Gholizadeh and Yang‐Xin^[^ ^94^ ^]^ corroborates these findings, showing that co‐doping graphene sheets results in a shift of the bandgap. These earlier investigations align with our current observations, reinforcing the understanding that dopant incorporation significantly affects the electronic structure of graphene‐based materials. The Fermi energy for LIG is −6.87 eV, while for dopant‐rich LIG it is −7.34 eV, resulting in a Fermi energy shift of −0.47 eV after doping.

Figure 7i shows the electron density as 2D plot with averaged density through XY cross section. High carrier densities are observed in areas where nitrogen dopant associated with graphene defects is present. Low densities are also achieved in boron‐rich sections. Boron is responsible for enhancing the conductivity, while nitrogen‐rich defects are attributed to improved electron emission, further tailored by the sharpened topography of the BDNW‐rich regions.

The estimated electrostatic potential maps resemble the dumbbell‐shaped charge density structures reported for Q‐carbon,^[^ ^95^ ^]^ attributed to the 2*p* orbital of carbon atoms, which is also supported by the PDOS results. Moreover, the *sp* ^2^‐bonded carbon atoms with mid‐gap states in the *sp* ^3^ carbon‐rich regions, along with the enhanced spatial conductivity in the surrounding amorphous carbon region due to the *sp* ^2^ carbon‐rich network, contribute to the exceptional field‐emission properties.

## 3 Conclusion

The novel hybrid nanocarbon surfaces developed in this study exhibit effective incorporation and integration of boron‐doped diamond nanowalls (BDNW) in the porous structure of laser‐induced graphene (LIG), with major structural transformations occurring during the lasing process and partial migration of diamond‐rich particles into the graphene nanostructure. Lasing of BDNW‐decorated PI sheets resulted in the in‐situ formation of LIG‐BDNW hybrid nanoscribed nanosurfaces with developed topography, boosting porosity and creating sharp edges rich in nitrogen defects. BDNW particles are characterized by their sharp edges induced by dopants and faceted morphology originating from diamond cores. The improvement in graphitization was attributed to the presence of BDNWs during the photo‐thermal conversion of the PI sheet, due to increased absorbance of particles and localized thermal effects. The transition from *sp* ^2^ to *sp* ^3^ hybridized nanocarbons at the interface of BDNW particles and LIG simulates inorganic Janus nanostructures, in which each face possesses distinct physical properties. This results in enhanced electronic functionality of these anisotropic hybrid nanostructures, efficiently handling carrier transfer and emission. Due to the low doping level of the LIG, the enhancement is mainly related to an increase in the number of electrons. However, because of p‐type doping in BDNWs, charge carrier enhancement for LIG‐BDNW hybrid nanostructures likely originates from an increase in both electron and hole carriers, resulting in a 207% emission enhancement in the studied temperature range.

The highly conductive surfaces tailored with BDNWs increase the emission sites of the LIG and facilitate charge transformation. Combined with the stability of BDNWs, this resulted in enhanced micro‐plasma ignition (μPI) properties with prolonged lifetime. The higher electrical conductivity of cathode materials results in less Ohmic drop and the development of higher electrical fields across the electrodes. Additionally, sharp‐edged nanostructures like graphene nanoplatelets and faceted BDNWs are prone to develop stronger local electric fields. Enhanced electrical conductivity in LIG‐BDNW hybrids aids the development of stronger electrical fields across cathode materials. Strong electric fields at the edges of both LIGs and BDNWs enhance the electron energy level, resulting in the formation of hot carriers. Spectroscopic analysis indicated the high quality of the resulting LIG, with carbon atoms predominantly exhibiting *sp* ^2^ hybridization. The observed broadening of the *π*‐band in the C1*s* X‐ray absorption spectrum is logically associated with the splitting of the *π*2*p*_z_ conduction subband due to slight corrugation of flat single‐layer graphene and a decrease in symmetry in the LIG, caused by a small number of defects in the graphene layers. Transitions of N1*s* electrons to free 2*p* states of nitrogen atoms were observed in areas of nitrogen‐doped graphene, with the formation of π*(C=N) and π*(pyridine‐N‐oxide) bonds, respectively. The incorporation of nitrogen and boron into graphene sheets has been explored as a feasible strategy to modify their electronic characteristics, as these dopants have atomic sizes comparable to that of carbon. These dopants can be used to engineer the bandgap and enhance the catalytic activity of graphene. Extended ab‐initio simulations supported the experimental findings, revealing that high carrier densities are observed in areas where nitrogen dopant associated with graphene defects is present. Low densities are also achieved in boron‐rich sections, with boron being responsible for enhancing the conductivity.

The scalable and rapid manufacturability of LIGs on flexible PI tapes with arbitrary patterns enables the fabrication of cathodes with customized geometries for μPI devices at a reasonable cost. Accordingly, hybrid nanomaterials based on LIG and conductive diamond nanostructures show great promise in the field of μPI devices, offering enhanced performance and manufacturing flexibility for next‐generation micro‐plasma ignition technologies for field‐emission displays, novel cold cathode‐based electronic devices and field‐emission microscopes.

## 4 Experimental Section

4.1

4.1.1

##### Fabrication of LIG‐BDNW Hybrid Nanostructures

The fabrication of the LIG‐BDNW hybrids started with the fabrication of BDNW films. A monocrystalline diamond substrate (MSY 0.75–1.25 (Microdiamant AG, USA)) was subjected to a microwave plasma‐enhanced chemical vapor deposition (MWPECVD) system (SEKI Technotron AX5400S from Japan) to grow the diamond nanowalls.^[^ ^96^, ^97^ ^]^ The growth temperature was maintained at 700 °C for 2 h. The doping level of boron in the gaseous phase, the [B]/[C] ratio, was maintained at 2000 ppm using diborane (B_2_H_6_) as the dopant precursor. The BDNW‐decorated PI sheets were subjected to direct laser writing using a CO_2_ laser (30 W laser system with *λ* = 10.6 μm) to prepare the LIG‐BDNW hybrids. The laser parameters were kept constant at 50% of the laser power and a 300 mm s^−1^ laser scan rate. The laser power was optimized to 50% because graphene formation was not observed at a power level below 50%, owing to insufficient energy for efficient photothermal conversion. Conversely, when the power was increased beyond the optimized power, the sample was partially burned, and ash formation was observed.^[^ ^98^ ^]^ Similarly, the scan speed was optimized at 300 mm s^−1^, ensuring the quality of the graphene. The pristine LIG nanostructures were fabricated without any BCNW particles for reference purposes. The optimization of laser operational conditions was performed in our team's previous studies.^[^ ^99^, ^100^ ^]^

##### Hall Measurements

The electrical conductivity, mobility, and carrier density of the films were estimated through Hall effect measurements conducted with the van der Pauw configuration (ECOPIA HMS‐3000) from RT to 573 K, in a 0.55 T magnetic field.

##### Field Electron Emission

The FEE performance of the LIG‐BDNW hybrids was evaluated by placing a hybrid in a high‐vacuum chamber with pressure below 10^−6^ Torr. The FEE experiments were carried out using a custom‐made, adjustable parallel plate capacitor setup, where a molybdenum (Mo) rod with a diameter of 2 mm was used as the anode and the LIG‐BDNW films as the cathode. A digital micrometer was used to measure the anode‐sample distance.

The current–voltage (*I–V)* characteristics were obtained using an electrometer (Keithley 2470) under a pressure below 10^−6^ Torr. The FEE properties of the materials were analyzed using the Fowler–Nordheim (F–N) theory^[^ ^42^ ^]^ $J_{e}=\left(\frac{A\beta^{2}E^{2}}{\varphi }\right)\exp\left(-\frac{B\varphi^{\frac{3}{2}}}{\beta E}\right)$; where *A* = 1.54 × 10^−6^ A eV V^−2^, *B* = 6.83 × 10^9^ eV^−3/2^ V m^−1^, *J*_*e*_ is the FEE current density, *E* is the applied field, *β* is the field‐enhancement factor, and *φ* is the work function of the emitting materials. The turn‐on field (*E*_0_) was designated as the point of intersection of the straight lines extrapolated from the low‐ and the high‐field segments of the F–N plots, namely, ln(*J*_e_/*E* ^2^)‐1/*E* plots.

##### Plasma Illumination

The characteristics of plasma illumination were studied using a microcavity, where the anode was made of indium tin oxide (ITO)‐coated glass, and the cathode consisted of LIG‐BDNW hybrids. A 1.0 mm thick Teflon spacer separated the anode and cathode, with an 8.0 mm diameter circular hole forming the microcavity. Plasma was initiated in bipolar pulse mode using a pulsed DC voltage at an Ar gas pressure of 1.3 × 10^4^ Pa. The plasma was observed using a USB microscope, and a Keithley 2470 electrometer was used to measure the current–voltage characteristics.

##### X‐Ray Absorption Spectroscopy

The high‐resolution C1*s*, N1*s* and O1*s* X‐ray absorption spectra of the LIG and LIG‐BDNW samples were measured using the facilities of the HE‐SGM beamline (HE‐SGM) at the BESSY II synchrotron radiation source of Helmholtz‐Zentrum Berlin (HZB).^[^ ^70^, ^101^ ^]^ The spectra were acquired under ultrahigh vacuum conditions (*P *≈ 1 × 10^−9^ Torr) at *T* = 300 K. The NEXAFS spectra were obtained by recording the total electron yield (TEY) using the PEY/TEY detector. The monochromator energy resolution near the C1*s* absorption edge (*hv* ≈ 285 eV), the N1*s* absorption edge (*hv* ≈ 400 eV), and the O1*s* absorption edge (*hv* ≈ 530 eV) was ≈100 meV, ≈150 meV, and ≈200 meV, respectively. The size of the X‐ray spot on the sample was ≈1200 × 200 μm. The photon energies in the range of the fine structure of the C1*s* X‐ray absorption spectra were calibrated against the energy position of the *π*‐resonance in the C1*s* X‐ray absorption spectrum of HOPG (*hv *≈ 285.45 eV).^[^ ^102^ ^]^ The photon energies in the range of the fine structure of the N1*s* and the O1*s* X‐ray absorption spectra were calibrated against the energy positions of the Ti2*p* (459.0 eV) and F1*s* (683.9 eV) absorption spectra of K_2_TiF_6_.^[^ ^102^, ^103^ ^]^ No radiation damage to the LIG and LIG‐BDNW samples were observed during the experiments.

##### Photoelectron Spectroscopy

The survey, HR C1*s*, and HR O1*s* photoelectron spectra were acquired at *hν* = 730 eV using a Scienta R3000 electron energy analyzer (Scienta). The analyzer pass energy was set to 200 eV and 50 eV, respectively. The C1*s*, Au4*f* and valence band spectra of the reference samples (HOPG and Au) were measured to calibrate the analyzer work function. The detection angle was close to that of normal emission. To analyze the data, the spectra were fitted by the Gaussian/Lorentzian convolution functions using the UNIFIT software.^[^ ^74^, ^76^ ^]^ The simultaneous optimization of the background parameters was done during the deconvolution procedure.

##### Raman Spectroscopy

The Raman analysis was performed by means of a confocal microRaman spectrometer (InVia, Renishaw, UK) equipped with a 532 nm excitation laser (Ar ion laser). The laser power was reduced to 1% of the total 50 mW to avoid sample heating. The Raman spectra were recorded in the range of 100–3200 cm^−1^. Each sample was analyzed at five independent points. To analyze the results, Raman bands were fitted by the Lorentzian convolution functions using the OriginLab software.

##### Scanning Electron Microscopy

The surface morphologies were investigated using a scanning electron microscope (SEM; FEI Quanta FEG 250) with a 10 kV beam accelerating voltage and a secondary electron Everhart–Thornley detector (SE‐ETD) operating in high‐vacuum mode (pressure of 10^−4^ Pa).

##### High‐Resolution Transmission Electron Microscopy

The microstructures were characterized using high‐resolution transmission electron microscopy (HRTEM) with a JEM‐2200FS field‐emission microscope at an acceleration voltage of 200 kV.

##### DFT Simulation

The computational simulations were conducted using the Quantum‐V‐ATK‐2023.12 software suite from Synopsys.^[^ ^104^, ^105^ ^]^ Initial geometric optimization of the structures was performed using the Linear Broyden–Fletcher–Goldfarb–Shanno (L‐BFGS), including Van der Waals D3 (VdW) corrections.^[^ ^106^ ^]^ The optimization criteria were set with energy convergence thresholds of 0.05 eV Å^−1^ and pressure below 0.1 GPa. The structures were optimized in terms of bond lengths and angles, and also with respect to cell volume under atmospheric pressure conditions, using the machine learned M3GNET framework.^[^ ^107^ ^]^ The electronic properties were calculated using density functional theory (DFT) within the generalized gradient approximation (GGA), employing the Perdew–Burke–Ernzerhof (PBE) functional. The pseudopotentials were used from the PseudoDojo library^[^ ^108^ ^]^ obtaining a high‐quality description of the atomic orbitals. Two computational slab models were constructed to show the interfacial characteristics and the surface properties of BDNW‐LIG. The interface configuration comprised *sp* ^3^‐hybridized carbon in the central region and *sp* ^2^‐hybridized carbon at the periphery, facilitating the investigation of bulk interfacial parameters. The surface configuration featured *sp* ^3^‐hybridized carbon on the left domain and oxygen‐terminated *sp* ^2^‐hybridized carbon BDNW on the right domain, with an incorporated vacuum region to examine the edge properties of LIG‐BDNW. Both structural models were systematically doped with nitrogen and boron atoms in concentrations consistent with XPS data.

## Conflict of Interest

The authors declare no conflicts of interest.

## Author Contributions

**Mohsen Khodadadiyazdi**: methodology (equal); writing—original draft (equal). **Mateusz Ficek**: conceptualization (equal); investigation (equal); methodology (equal). **Maria Brzhezinskaya**: data curation (equal); formal analysis (equal); methodology (equal); visualization (equal); writing—original draft (equal). **Shradha Suman**: data curation (equal); visualization (equal). **Salila Kumar Sethy**: data curation (equal); formal analysis (equal); writing—original draft (equal). **Kamatchi Jothiramalingam Sankaran**: conceptualization (equal); data curation (equal); visualization (equal); writing—original draft (equal). **Bartłomiej Dec**: data curation (equal); writing—original draft (equal). **Mattia Pierpaoli**: funding acquisition (equal); visualization (equal). **Sujit Deshmukh**: visualization (equal); writing—original draft (equal). **Miroslaw Sawczak**: data curation (equal); writing—original draft (equal). **William A. Goddard III**: funding acquisition (equal); project administration (equal); supervision (equal); validation (equal). **Robert Bogdanowicz**: funding acquisition (equal); project administration (equal); supervision (equal); writing—review & editing (equal).

## Data Availability

The data that support the findings of this study are available from the corresponding author upon reasonable request.
